# Forward and Inverse Dynamics of a Six-Axis Accelerometer Based on a Parallel Mechanism

**DOI:** 10.3390/s21010233

**Published:** 2021-01-01

**Authors:** Linkang Wang, Jingjing You, Xiaolong Yang, Huaxin Chen, Chenggang Li, Hongtao Wu

**Affiliations:** 1 College of Mechanical and Electronic Engineering, Nanjing Forestry University, Nanjing 210037, China; wlk0710@njfu.edu.cn (L.W.); chx915147@njfu.edu.cn (H.C.); 2 School of Mechanical Engineering, Nanjing University of Science and Technology, Nanjing 210037, China; xiaolongyang@njust.edu.cn; 3 School of Mechanical and Electrical Engineering, Nanjing University of Aeronautics and Astronautics, Nanjing 210016, China; lichenggang@nuaa.edu.cn (C.L.); meehtwu@nuaa.edu.cn (H.W.)

**Keywords:** six-axis accelerometer, parallel mechanism, forward dynamics, inverse dynamics, decoupling

## Abstract

The solution of the dynamic equations of the six-axis accelerometer is a prerequisite for sensor calibration, structural optimization, and practical application. However, the forward dynamic equations (FDEs) and inverse dynamic equations (IDEs) of this type of system have not been completely solved due to the strongly nonlinear coupling relationship between the inputs and outputs. This article presents a comprehensive study of the FDEs and IDEs of the six-axis accelerometer based on a parallel mechanism. Firstly, two sets of dynamic equations of the sensor are constructed based on the Newton–Euler method in the configuration space. Secondly, based on the analytical solution of the sensor branch chain length, the coordination equation between the output signals of the branch chain is constructed. The FDEs of the sensor are established by combining the coordination equations and two sets of dynamic equations. Furthermore, by introducing generalized momentum and Hamiltonian function and using Legendre transformation, the vibration differential equations (VDEs) of the sensor are derived. The VDEs and Newton–Euler equations constitute the IDEs of the system. Finally, the explicit recursive algorithm for solving the quaternion in the equation is given in the phase space. Then the IDEs are solved by substituting the quaternion into the dynamic equations in the configuration space. The predicted numerical results of the established FDEs and IDEs are verified by comparing with virtual and actual experimental data. The actual experiment shows that the relative errors of the FDEs and the IDEs constructed in this article are 2.21% and 7.65%, respectively. This research provides a new strategy for further improving the practicability of the six-axis accelerometer.

## 1. Introduction

The robustness of the control system can be improved by introducing acceleration feedback in the robot control system [1]. The motion of an object in space is generally a six-degree-of-freedom motion. Therefore, the performance of the control system can be further improved by using the six-axis accelerometer for feedback control of the robot. In addition, fields such as navigation [2], consumer electronics [3], flight vehicles [4], and biomechanics [5] also require the simultaneous measurement of the six spatial components of the acceleration. At present, most of the fields mentioned above use inertial measurement units (IMUs) to measure the space six-axis accelerations of an object, that is, a combined measurement scheme of three linear accelerations and three gyroscopes [6,7]. In general, low-cost IMUs are often limited by gyroscope defects, such as large size, high cost, and large bias instability. Accelerometers with high precision and high reliability can be manufactured at a lower price. Therefore, it is interesting to use a combination of accelerometers to detect space six-axis accelerations [8].

Tan et al. [9] analyzed and gave two prerequisites for the combined six-axis accelerometer, namely that the configuration matrix of the elastic body is reversible, and at least 6 accelerometers are required. Based on the above two conditions, a large number of scholars have proposed and studied space six-axis accelerations measurement schemes based on the configuration of the elastic body and the number of accelerometers. The types of elastomer configurations are mainly divided into cubic configuration [8,9,10], tetrahedral configuration [11], and cylindrical configuration [12]. The number of utilized linear accelerometer is 6 [9,10], 8 [12], 9 [13], 12 [8,14]. The above combination scheme based on the accelerometer avoids using a gyroscope with a complicated structure and difficult maintenance and reduces the cost of the sensor. However, its engineering realization is more difficult due to the complicated topology and high installation accuracy. In addition, this type of sensor has an inner lever-arm effect [15]. The smaller the size, the more obvious the effect, which makes it difficult to achieve miniaturization of the sensor. The above-mentioned defects can be solved by integrating the inertial units of the sensor into a whole, that is, the sensor has only one inertial unit. Lv et al. [16] proposed and studied a six-axis accelerometer based on six sets of quartz crystal sets and analyzed the linear mapping between inputs and outputs. Meng et al. [17] proposed a six-axis accelerometer with a dual annular membrane structure, and used ANSYS software to determine the corresponding relationship between the deformation of the diaphragm and the mass inertial force. Ranjith et al. [18] designed a piezoresistive six-axis accelerometer and calculated the sensitivity characteristics through calibration experiments. Although the above scheme proposes a six-axis acceleration measurement scheme based on a single mass, it is difficult to accurately establish the dynamic equations of the system theoretically. Since Gaillet and Reboulet began to measure the space six-axis forces with the parallel mechanism in 1983 [19], many scholars have proposed the measurement scheme of space six-axis forces based on the parallel mechanism [20,21,22,23,24]. The six-axis force sensor based on the Stewart platform generally has the characteristics of high stiffness and load capacity through distributing the loading axially over the six legs [25]. Inspired by the design scheme of the six-axis force sensor based on the Stewart platform, some scholars proposed and studied the six-axis acceleration measurement scheme that only contained one inertial unit by using the Stewart platform as the elastic body of the sensor [26,27,28,29,30]. Therefore, this article will take the six-axis accelerometer proposed in Reference [31] as the research object.

Considering the dynamic equation of the six-axis accelerometer, the process of deriving the outputs (i.e., sensing unit signal) from the inputs (i.e., measurement signal) is called “forward dynamics”; on the contrary, the process of inferring the inputs based on the outputs is called “inverse dynamics”. The relationship between the measurement signal of the branch chain and the measurement signal can be determined by analyzing the FDEs of the sensor, which provide a theoretical basis for the calibration of the sensor [32]. In addition, the FDEs are also a prerequisite for structural optimization and fault-tolerant processing of sensors [33,34,35]. Since the FDEs of the sensor are a statically indeterminate problem, it generally needs to be solved by a supplementary equation constructed by the forward kinematic equation of the elastic body and the geometric coordination equation. However, the forward kinematics of the parallel mechanism itself is the difficulty of the mechanism [36], which leads to few reports on the FDEs of the six-axis accelerometer. You et al. [37] analyzed the FDEs of the sensor based on the kinematic equation of the sensor elastic body and the geometric constraint relationship of the spherical hinge. However, due to the influence of the kinematic equation, the FDEs have the defects of complex modeling process and low solution efficiency, and the modeling method is not universal. Raoofian et al. [38] and Yuan et al. [39] modeled and analyzed the FDEs of the parallel mechanism by using the Lagrange formulation. This method faces the problem of high computational load due to the combination of differential equations and algebraic equations used in the modeling process. Based on the above analysis, this article, on the premise of avoiding the use of sensor forward kinematics, formulates the FDEs of the sensos by constructing the output signal supplementary equations and combining the Newton–Euler equations.

The solution of the IDEs of the six-axis accelerometer is also called the decoupling algorithm of the sensor. Since the six-axis force sensor has determined the direction of the branch chain during the calibration stage, and this direction will not change during the measurement process, thereby only the axial force of each branch is required for decoupling [40]. However, the direction of the branch chain of the six-axis accelerometer is changing during the measurement process, so the axial force and direction of the branch chain are required for decoupling calculation. This shows that the relationship between the inputs and outputs of the six-axis accelerometer is constantly changing, and this relationship is related to the acceleration to be measured. Therefore, the solution of the IDEs of the six-axis accelerometer involves the coupled calculation of all output quantities at continuous moments. However, the measurement value of the six-axis force sensor is only related to the output at the current moment. For real-time applications of the Stewart type of six-axis accelerometers, it becomes indispensable to decouple three linear accelerations from three angular accelerations. References [26] and [41] ignore the nonlinear coupling terms (the amount of rotation of the pedestal) in the equation when dealing with the IDEs of the sensor. Although this scheme improves the efficiency of solving the IDEs, the accuracy of the equation solution is reduced. Xia et al. [30] proposed a method for modeling the IDEs of a six-axis accelerometer based on the Kane’s dynamics equation. However, this scheme introduces more kinematics items, which leads to the real-time performance of the algorithm cannot be guaranteed. You et al. [31] studied the IDEs of a six-axis accelerometer based on the Newton–Euler method. This scheme derives the explicit recursive formula of key feature quantities, but the acceleration expressions are highly nonlinear with complex structures. At the same time, the physical meaning of the intermediate parameter (generalized coordinates) in the equation is not clear. In order to solve the above problems, this article constructs the VDEs of the sensor with respect to the intermediate parameters by introducing generalized momentum and Hamiltonian equations. Then, the IDEs of the sensor are constructed by combining the Newton–Euler equations.

The remainder of this article is organized as follows: After the introduction, Section 2 concerns the structure and measurement principle of the proposed six-axis accelerometer and describes the sensor’s spherical hinge arrangement and the coordinate relationship of the elastic body. Section 3 introduces the modeling process of the sensor’s dynamic equations. On the one hand, the coordination relationship of the output signal of the sensor is analyzed, and the FDEs of the sensor is constructed based on this. On the other hand, based on the Newton–Euler equation and Hamiltonian equation, the IDEs of the sensor are analyzed, and the solution process of the IDEs that can realize the real-time decoupling of the measured acceleration is given. Section 4 and Section 5 describes the virtual experiments and actual experiments to validate the practicability of the proposed six-axis accelerometer. Finally, Section 6 provides the conclusions.

## 2. Structural Design and Principle Model

This section presents the structural design and principle model for a six-axis accelerometer that was utilized to obtain the desired output of the accelerations.

### 2.1. Structural Design

**Figure 1:**
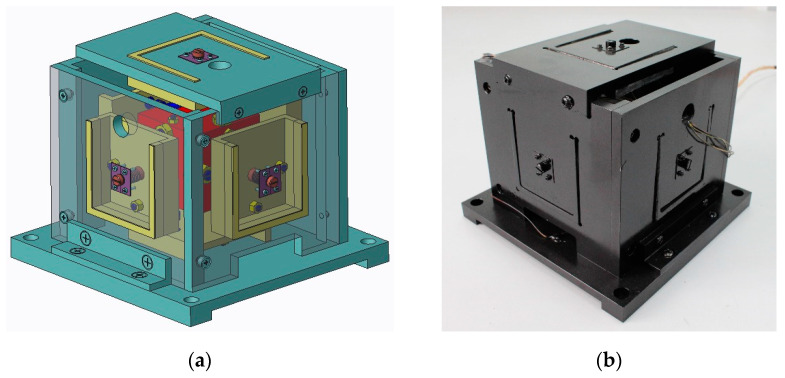
Prototype of the six-axis accelerometer: (**a**) Digital prototype of six-axis accelerometer; (**b**) Physical prototype of six-axis accelerometer.

**Figure 2:**
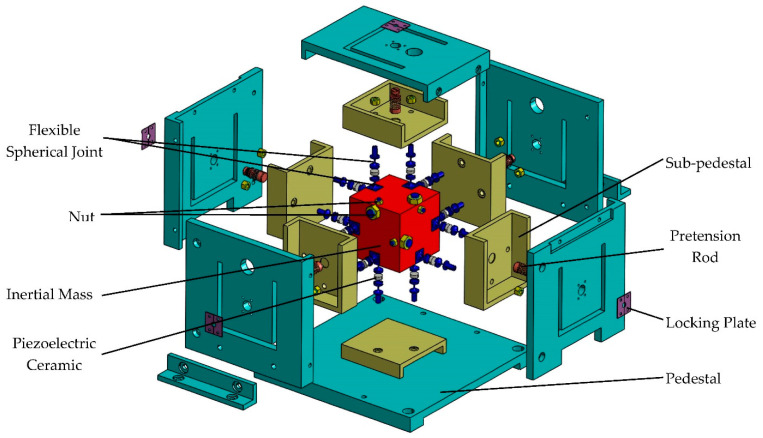
The exploded 3-D drawing of the six-axis accelerometer.

**Figure 3:**
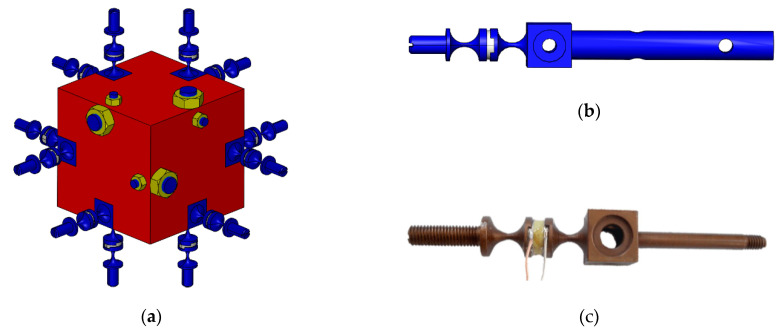
(**a**) Configuration scheme of the branch chain combination; (**c**) Structure model of a branch chain; (**b**) Physical prototype of a branch chain.

The digital and physical prototype of the six-axis accelerometer is shown in Figure 1a,b, respectively. In order to display the assembly model of the six-axis accelerometer and the distribution of internal branches more intuitively, the exploded 3-D drawing is shown in Figure 2. The six-axis accelerometer is composed of a cube-shaped inertia mass, a cube-shaped pedestal, a sub-pedestal, a locking plate, a pretension rod, and twelve SPS (S—prismatic; P—prismatic pair) branch chains connecting the inertia mass and the pedestal. The configuration relationship between the branch chain combinations is shown in Figure 3a. The structure model and physical prototype of a single SPS branch chain are shown in Figure 3b,c, respectively. Each SPS branch chain consists of two flexible spherical joints [22,42] and a piezoelectric ceramic in series. The piezoelectric ceramics are cylindrical, both ends of which are connected with flexible spherical joints by epoxy resin adhesive.

In practice, the pedestal of the accelerometer is mounted on a moving carrier, e.g., robot, aircraft, and the human body. When the external six-dimensional acceleration acts on the sensor, the inertial mass will produce a certain posture change under the action of inertial force, so the branch chain will be stressed. Piezoelectric ceramics will generate electrical signals due to the axial force of the branch chain. In this article, we define the positive direction of the axial force pointing from the sub-pedestal to the inertial mass.

### 2.2. Principle Model and Its Coordinate System

**Figure 4:**
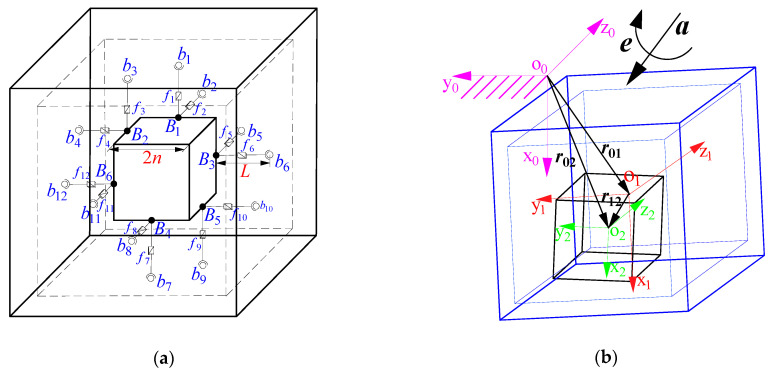
Principle model and coordinate system: (**a**) principle model of the six-axis accelerometer; (**b**) Coordinate relationships between inertial mass, pedestal and ground.

Figure 4a shows the principle model of the accelerometer, which is a 12-6 Stewart derivative parallel mechanism. The pedestal, inertial mass, and piezoelectric ceramics can be regarded as a base, a moving platform, and sliding pairs in the parallel mechanism, respectively. *b_i_* (*i* = 1, 2, …, 12) and *B_j_* (*j* = 1, 2, …, 6) are the spherical joints fixed on the pedestal and the inertia mass, respectively. *f_i_* (*i* = 1, 2, …, 12) are the forces along the axis of the branch chains. 2*n* denotes the edge-length of the inertia mass, and *L* denotes the initial length of the 12 branches.

As shown in Figure 4b, the coordinate systems O_0_{x_0_, y_0_, z_0_}({O_0_}), O_1_{x_1_, y_1_, z_1_}({O_1_}), and O_2_{x_2_, y_2_, z_2_}({O_2_}) are attached to the ground, the center O_1_ of the pedestal, and the center O_2_ of the inertial mass, respectively. The x-axis is parallel with *B*_4_*b*_7_, and y-axis is parallel with *B*_2_*b*_4_. The z-axis is determined by the right-hand rule. The origins and coordinate axes of the three coordinate systems coincide respectively at the initial time. The position vectors between the three coordinate systems are represented by ***r***_01_, ***r***_12_, ***r***_02_, respectively. It should be pointed out here that due to the high rigidity of the sensor branch chains and the extremely small deformation of the piezoelectric ceramic, the relative pose between the pedestal and inertial mass is enlarged for clear explanation. ***a*** and ***e*** are linear acceleration and angular acceleration, respectively, that are applied on the pedestal.

## 3. Dynamic Analysis of the System

The inputs of the system are 6 independent components of ***a*** and ***e***, and the outputs are the axial forces of 12 branches. The dynamic model of the system is set up based on the Newton–Euler Equations.

(1) $$\left[\begin{array}{c} a\\ 0 \end{array}\right]=\frac{1}{m}R_{01}F_{a}+{\left[\begin{array}{cccc} g & 0 & 0 & 0 \end{array}\right]}^{T}$$

(2) $$\left[\begin{array}{c} e\\ 0 \end{array}\right]=\frac{3}{2mn}R_{01}F_{e}$$

where $F_{a}=\left[\begin{array}{c} f_{1}+f_{3}-f_{7}-f_{9}\\ -f_{4}+f_{6}+f_{10}-f_{12}\\ -f_{2}-f_{5}+f_{8}+f_{11}\\ 0 \end{array}\right]$, $F_{e}=\left[\begin{array}{c} f_{5}-f_{6}+f_{11}-f_{12}\\ f_{1}-f_{2}+f_{7}-f_{8}\\ -f_{3}+f_{4}-f_{9}+f_{10}\\ 0 \end{array}\right]$, *m* denotes the mass of the inertial mass, g is the gravitational acceleration. ***R***_01_ denotes the rotation matrix that the reference {O_1_} is relative to the {O_0_}. In this article, quaternion ***∧*** = *λ*_0_ + *λ*_1_***i*** + *λ*_2_***j*** + *λ*_3_***k*** with one real part *λ*_0_ and three imaginary parts *λ*_1_, *λ*_2_, *λ*_3_ are employed to express the rotation matrix as

(3) $$R_{01}={\left(\lambda^{-}\right)}^{T}\lambda^{+}$$

where

$$\lambda^{+}=\;\left[\begin{array}{cccc} \lambda_{0} & -\lambda_{3} & \lambda_{2} & \lambda_{1}\\ \lambda_{3} & \lambda_{0} & -\lambda_{1} & \lambda_{2}\\ -\lambda_{2} & \lambda_{1} & \lambda_{0} & \lambda_{3}\\ -\lambda_{1} & -\lambda_{2} & -\lambda_{3} & \lambda_{0} \end{array}\right],\;\lambda^{-}=\left[\begin{array}{cccc} \lambda_{0} & \lambda_{3} & -\lambda_{2} & \lambda_{1}\\ -\lambda_{3} & \lambda_{0} & \lambda_{1} & \lambda_{2}\\ \lambda_{2} & -\lambda_{1} & \lambda_{0} & \lambda_{3}\\ -\lambda_{1} & -\lambda_{2} & -\lambda_{3} & \lambda_{0} \end{array}\right]$$

For a unit quaternion

(4) $$u=\lambda_{0}^{2}+\lambda_{1}^{2}+\lambda_{2}^{2}+\lambda_{3}^{2}=1$$

Based on Equations (1) and (2), it can be seen that the inputs and outputs of the six-axis accelerometer based on the parallel mechanism are strongly nonlinearly coupled, and the transformation matrix ***R***_01_ is constantly changing. Therefore, the decoupling calculation (solution of IDEs) of this sensor is more complicated than the six-axis force sensor based on the parallel mechanism [21,23]. To ensure the accuracy of the analysis and reduce the complexity of modeling, the following assumptions are made:

(1) Inertial mass, pedestal and sub-pedestals are all rigid bodies with no deformation. The weight of the spherical joints and piezoelectric ceramics can be ignored because the inertial mass has a large mass. Therefore, all branches are regarded as ideal two-force rod components [42].
(2) In a multi-axis sensor system, the negative impact of the flexible spherical joint can be ignored due to the large axial stiffness of the branch chain [22]. Therefore, all flexible spherical joints are assumed to be ideal without friction and creep.
(3) For a six-axis accelerometer system, the damping force can be ignored due to the greater stiffness of the system [12,28].

### 3.1. Forward Dynamics

From Equations (1) and (2), the mapping relationship between outputs and inputs can be expressed as:

(5) $$F_{a}=mR_{01}^{T}\left(\left[\begin{array}{c} a\\ 0 \end{array}\right]-{\left[\begin{array}{cccc} g & 0 & 0 & 0 \end{array}\right]}^{T}\right)$$

(6) $$F_{e}=\frac{2mn}{3}R_{01}^{T}\left[\begin{array}{c} e\\ 0 \end{array}\right]$$

The problem of solving Equations (5) and (6) is a second-order statically indeterminate problem because the number of unknowns is more than the number of equations. A common strategy used to solve the above problem is to establish a supplementary equation.

In {O_1_}, the vectors ***b****_i_*_,1_ of points *b_i_* are written as

(7) $$\begin{array}{l} \left[b_{1,1},b_{2,1},b_{3,1},b_{4,1},b_{5,1},b_{6,1},b_{7,1},b_{8,1},b_{9,1},b_{10,1},b_{11,1},b_{12,1}\right]=\\ \left[\begin{array}{cccccccccccc} -n-L & -n & -n-L & -n & 0 & 0 & n+L & n & n+L & n & 0 & 0\\ 0 & 0 & n & n+L & -n & -n-L & 0 & 0 & -n & -n-L & n & n+L\\ n & n+L & 0 & 0 & n+L & n & -n & -n-L & 0 & 0 & -n-L & -n \end{array}\right] \end{array}$$

In the {O_2_}, the vectors ***B**_j_*_,2_ of points ***B****_j_* can be written as:

(8) $$\left[B_{1,2},B_{2,2},B_{3,2},B_{4,2},B_{5,2},B_{6,2}\right]=\left[\begin{array}{cccccc} -n & -n & 0 & n & n & 0\\ 0 & n & -n & 0 & -n & n\\ n & 0 & n & -n & 0 & -n \end{array}\right]$$

The coordinate mapping formula of the coordinate of spherical joints *B_j_* from the reference {O_2_} to the {O_1_} is shown as follows:

(9) $$\left[\begin{array}{c} B_{j,1}\\ 0 \end{array}\right]=\left[\begin{array}{c} r_{12}\\ 0 \end{array}\right]+R_{12}\left[\begin{array}{c} B_{j,2}\\ 0 \end{array}\right]$$

where ***r***_12_ = (x, y, z)^T^, ***R***_12_ is expressed by quaternion ***Φ*** = *φ*_0_ + *φ*_1_***i*** + *φ*_2_***j*** + *φ*_3_***k*** representing the rotation matrix from {O_2_} to {O_1_}.

In our prophase research, we have found that the second-order and above small amount of the pose parameter of the inertia mass relative to the pedestal can be ignored since the inertia mass moves slightly relative to the pedestal [31]. Therefore, ***R***_12_ can be expressed as:

(10) $$R_{12}=\left[\begin{array}{cccc} 1 & -2\varphi_{0}\varphi_{3} & 2\varphi_{0}\varphi_{2} & 0\\ 2\varphi_{0}\varphi_{3} & 1 & -2\varphi_{0}\varphi_{1} & 0\\ -2\varphi_{0}\varphi_{2} & 2\varphi_{0}\varphi_{1} & 1 & 0\\ 0 & 0 & 0 & 1 \end{array}\right]$$

Then, the kinematic Equation of the branch chains is expressed as:

(11) $$l_{i}\left[\begin{array}{c} e_{i,1}\\ 0 \end{array}\right]=\left[\begin{array}{c} b_{i,1}\\ 0 \end{array}\right]-\left[\begin{array}{c} B_{j,1}\\ 0 \end{array}\right]=\left[\begin{array}{c} b_{i,1}\\ 0 \end{array}\right]-\left(\left[\begin{array}{c} r_{12}\\ 0 \end{array}\right]+R_{12}\left[\begin{array}{c} B_{j,2}\\ 0 \end{array}\right]\right)\;,\;j=\left\{ \begin{array}{l} 1\;\left(i=1,2\right);\;4\;\left(i=7,8\right);\\ 2\;\left(i=3,4\right);\;5\;\left(i=9,10\right);\\ 3\;\left(i=5,6\right);\;6\;\left(i=11,12\right); \end{array}\right.$$

where *l_i_* represents the actual length of the *i*th branch and ***e****_i_*_,1_ represents the directional vector of the *i*th branch in {O_1_}.

According to Equation (11), the length of the branch can be expressed as:

(12) $$l_{i}=\sqrt{{\left[l_{i}e_{i}\right]}^{T}\left[l_{i}e_{i}\right]}$$

Expand Equation (12) according to Taylor formula, and ignore small quantities of second order and above

(13) $$l_{i}\approx L+\frac{1}{2L}\left({\left[l_{i}e_{i}\right]}^{T}\left[l_{i}e_{i}\right]-L^{2}\right)$$

Substituting Equations (7), (8), (10) into (11) and combining Equation (13), *l*_1_–*l*_12_ are expressed, respectively, as follows:

(14) $$\{\begin{array}{c} l_{1}\approx L-x-2n\varphi_{0}\varphi_{2};\begin{array}{c} l_{2}\approx L+z+2n\varphi_{0}\varphi_{2};l_{3}\approx \begin{array}{c} L-x+2n\varphi_{0}\varphi_{3}; \end{array}l_{4}\approx \begin{array}{c} L+y-2n\varphi_{0}\varphi_{3} \end{array}; \end{array}\\ l_{5}\approx \begin{array}{c} L+z-2n\varphi_{0}\varphi_{1} \end{array};\begin{array}{c} l_{6}\approx \begin{array}{c} L-y+2n\varphi_{0}\varphi_{1} \end{array};l_{7}\approx \begin{array}{c} \begin{array}{c} L+x-2n\varphi_{0}\varphi_{2} \end{array}; \end{array}l_{8}\approx \begin{array}{c} L-z+2n\varphi_{0}\varphi_{2} \end{array}; \end{array}\\ l_{9}\approx \begin{array}{c} L+x+2n\varphi_{0}\varphi_{3} \end{array};\begin{array}{c} l_{10}\approx \begin{array}{c} L-y-2n\varphi_{0}\varphi_{3} \end{array};l_{11}\approx \begin{array}{c} \begin{array}{c} L-z-2n\varphi_{0}\varphi_{1} \end{array}; \end{array}l_{12}\approx \begin{array}{c} \begin{array}{c} L+y+2n\varphi_{0}\varphi_{1} \end{array} \end{array}. \end{array} \end{array}$$

According to Hooke’s law, the length of the *i*th branch can also be expressed as:

(15) $$l_{i}\approx L-\frac{f_{i}}{k_{i}}$$

where *k_i_* represent the stiffness of the *i*th branch.

Combining Equations (14) and (15), the supplementary equation for the branched chain axial force can be expressed as follows:

(16) $$\{\begin{array}{c} f_{1}/k_{1}-f_{3}/k_{3}-f_{7}/k_{7}+f_{9}/k_{9}=0\\ f_{4}/k_{4}+f_{6}/k_{6}-f_{10}/k_{10}-f_{12}/k_{12}=0\\ f_{2}/k_{2}-f_{5}/k_{5}-f_{8}/k_{8}+f_{11}/k_{11}=0\\ f_{5}/k_{5}+f_{6}/k_{6}+f_{11}/k_{11}+f_{12}/k_{12}=0\\ \begin{array}{l} f_{1}/k_{1}+f_{2}/k_{2}+f_{7}/k_{7}+f_{8}/k_{8}=0\\ f_{3}/k_{3}+f_{4}/k_{4}+f_{9}/k_{9}+f_{10}/k_{10}=0 \end{array} \end{array}$$

Combining Equations (5), (6), (16), the relationship between the axial force and the acceleration of the six-axis accelerometer can be obtained as follows:

(17) $$CF=D=\left[\begin{array}{c} mR_{01}^{T}{\left(\left[\begin{array}{c} a\\ 0 \end{array}\right]-{\left[\begin{array}{cccc} g & 0 & 0 & 0 \end{array}\right]}^{T}\right)}_{1\sim 3}\\ {\left(\frac{2mn}{3}R_{01}^{T}\left[\begin{array}{c} e\\ 0 \end{array}\right]\right)}_{1\sim 3}\\ 0_{6\times 1} \end{array}\right]$$

where (●)*_k_* represents the *k*th element of the vector, ***F*** = [*f*_1_,*f*_2_,*f*_3_,*f*_4_,*f*_5_,*f*_6_,*f*_7_,*f*_8_,*f*_9_,*f*_10_,*f*_11_,*f*_12_]^T^, ***0***_6×1_ = [0,0,0,0,0,0]^T^,

$$C=\left[\begin{array}{cccccccccccc} 1 & 0 & 1 & 0 & 0 & 0 & -1 & 0 & -1 & 0 & 0 & 0\\ 0 & 0 & 0 & -1 & 0 & 1 & 0 & 0 & 0 & 1 & 0 & -1\\ 0 & -1 & 0 & 0 & -1 & 0 & 0 & 1 & 0 & 0 & 1 & 0\\ 0 & 0 & 0 & 0 & 1 & -1 & 0 & 0 & 0 & 0 & 1 & -1\\ 1 & -1 & 0 & 0 & 0 & 0 & 1 & -1 & 0 & 0 & 0 & 0\\ 0 & 0 & -1 & 1 & 0 & 0 & 0 & 0 & -1 & 1 & 0 & 0\\ 1/k_{1} & 0 & -1/k_{3} & 0 & 0 & 0 & -1/k_{7} & 0 & 1/k_{9} & 0 & 0 & 0\\ 0 & 0 & 0 & 1/k_{4} & 0 & 1/k_{6} & 0 & 0 & 0 & -1/k_{10} & 0 & -1/k_{12}\\ 0 & 1/k_{2} & 0 & 0 & -1/k_{5} & 0 & 0 & -1/k_{8} & 0 & 0 & 1/k_{11} & 0\\ 0 & 0 & 0 & 0 & 1/k_{5} & 1/k_{6} & 0 & 0 & 0 & 0 & 1/k_{11} & 1/k_{12}\\ 1/k_{1} & 1/k_{2} & 0 & 0 & 0 & 0 & 1/k_{7} & 1/k_{8} & 0 & 0 & 0 & 0\\ 0 & 0 & 1/k_{3} & 1/k_{4} & 0 & 0 & 0 & 0 & 1/k_{9} & 1/k_{10} & 0 & 0 \end{array}\right]$$

Since matrices ***C*** are 12 × 12 full-rank matrices, ***F*** has a unique solution:

(18) $$F=C^{-1}D$$

### 3.2. Inverse Dynamics

#### 3.2.1. Equation Establishment

The research results of Reference [37] show that the relative motion parameters between the inertial mass and the pedestal can be ignored when constructing the IDEs of the sensor. Therefore, ***R***_12_ is regarded as the identity matrix when analyzing the IDEs in this section, that is, ***R***_02_ is equivalent to ***R***_01_.

Establishing the system dynamic model:

(19) $$\frac{d}{dt}\left(\frac{\partial T}{\partial {\dot{s}}_{j}}\right)-\frac{\partial T}{\partial s_{j}}=Q_{j}+\mu \frac{\partial u}{\partial s_{j}}\left(j=1\sim 7\right)$$

where *T* is the kinetic energy function of the system that contains the translational and rotational kinetic energy, *Q_j_* is the generalized force of the system, *μ* is the lagrange undetermined multiplier, and *s_j_* is the generalized coordinate of the system. The first three generalized coordinates *s*_1_, *s*_2_ and *s*_3_ are set to be the three components of ***r***_01_, and the next four generalized coordinates *s*_4_, *s*_5_, *s*_6_ and *s*_7_ are set to be the parts of ***∧***, corresponding to *λ*_1_, *λ*_2_, *λ*_3,_ and *λ*_0_ respectively. Let $s_{T}={\left[s_{1},s_{2},s_{3}\right]}^{T}$, $s_{R}={\left[s_{4},s_{5},s_{6},s_{7}\right]}^{T}$.

The sum of the kinetic energy of the system is:

(20) $$T=\frac{1}{2}m{\left[\begin{array}{c} {\dot{r}}_{02}\\ 0 \end{array}\right]}^{T}\left[\begin{array}{c} {\dot{r}}_{02}\\ 0 \end{array}\right]+\frac{1}{2}{\left[\begin{array}{c} \omega_{02}\\ 0 \end{array}\right]}^{T}R_{01}I_{2}R_{01}^{T}\left[\begin{array}{c} \omega_{02}\\ 0 \end{array}\right]$$

where ${\dot{r}}_{02}$ and $\omega_{02}$ are the absolute linear velocity and angular velocity, respectively. ***I***_2_ is the generalized inertia mass matrix expressed {O_2_}.

Considering the relationship between the angular velocity and rotation matrix, ***ω***_02_ can be expressed in quaternions as:

(21) $$\left[\begin{array}{c} \omega_{02}\\ 0 \end{array}\right]=2{\left(\lambda^{-}\right)}^{T}{\dot{s}}_{R}$$

***I***_2_ can be written as a diagonal matrix.

(22) $$I_{2}=\frac{2}{3}mn^{2}\mathrm{diag}\left(1,1,1,1\right)$$

Substituting Equations (21), (22) into (20), *T* can be rewritten as:

(23) $$T=\frac{1}{2}m{\left({\dot{s}}_{T}\right)}^{T}{\dot{s}}_{T}+\frac{4}{3}mn^{2}{\left({\dot{s}}_{R}\right)}^{T}{\dot{s}}_{R}$$

The generalized force *Q_j_* including gravitational potential energy can be expressed as:

(24) $$Q_{j}=\sum_{j=1}^{6}\left(R_{01}\sum_{k=2j-1}^{2j}\left(f_{k}\left[\begin{array}{c} e_{k,1}\\ 0 \end{array}\right]\right)\cdot \frac{\partial }{\partial s_{j}}\left[\left[\begin{array}{c} r_{01}\\ 0 \end{array}\right]+R_{01}\left[\begin{array}{c} B_{j,2}\\ 0 \end{array}\right]\right]\right)+mg{\left[\begin{array}{ccc} 1 & 0 & 0 \end{array}\right]}^{T}\cdot \frac{\partial r_{01}}{\partial s_{j}}$$

Generalized momentum *p_j_* (*j* = 1,2,…,7) and Hamiltonian function *H* are introduced, according to the definition. Let $p_{T}={\left[p_{1},p_{2},p_{3}\right]}^{T}$, $p_{R}={\left[p_{4},p_{5},p_{6},p_{7}\right]}^{T}$.

(25) $$p_{j}=\frac{\partial T}{\partial {\dot{s}}_{j}}$$

(26) $$H=\sum_{j=1}^{7}p_{j}{\dot{s}}_{j}-T$$

Substituting Equation (23) into (25) yields:

(27) $${\dot{s}}_{T}=\frac{1}{m}p_{T}$$

(28) $${\dot{s}}_{R}=\frac{3}{8mn^{2}}p_{R}$$

It can be obtained that *H* is equal to *T* by substituting Equations (23), (25) into (26), and then combining Equations (27) and (28):

(29) $$H=\frac{1}{2m}{\left(p_{T}\right)}^{T}p_{T}+\frac{3}{16mn^{2}}{\left(p_{R}\right)}^{T}p_{R}$$

In phase space, according to Legendre transformation:

(30) $$\frac{\partial T}{\partial s_{j}}=-\frac{\partial H}{\partial s_{j}}$$

Substituting Equations (25), (30) into (19) yields:

(31) $${\dot{p}}_{j}=Q_{j}+\mu \frac{\partial u}{\partial s_{j}}-\frac{\partial H}{\partial s_{j}}\;(j=1\sim 7)$$

Substituting Equations (3), (4), (24), and (29) into (31) gives:

(32) $${\dot{p}}_{R}=2n\lambda^{+}\left[\begin{array}{l} f_{5}-f_{6}+f_{11}-f_{12}\\ f_{1}-f_{2}+f_{7}-f_{8}\\ -f_{3}+f_{4}-f_{9}+f_{10}\\ -\;\sum_{i=1}^{12}f_{i}+\mu /n \end{array}\right]$$

Find the first derivative of time on both sides of Equation (4). It can be obtained by combining Equation (28) so that there is an orthogonal relationship between generalized coordinates $s_{R}$ and generalized momentum $p_{R}$.

(33) $${\left(s_{R}\right)}^{T}p_{R}=0$$

Take the first derivative with respect to time on both sides of the Equation (33) and substitute it into the Equation (28):

(34) $${\left(s_{R}\right)}^{T}{\dot{p}}_{R}=-\frac{3}{8mn^{2}}{\left\|p_{R}\right\|}_{2}^{2}$$

where ${\left\|•\right\|}_{2}$ represents the Euclidean norm of the vector.

Equation (32) is multiplied by $s_{R}$ on both sides of the equal sign and combined with Equation (34), *μ* is figured out:

(35) $$\mu =n\sum_{i=1}^{12}f_{i}-\frac{3}{16mn^{2}}{\left\|p_{R}\right\|}_{2}^{2}$$

Substituting Equation (35) into (32) gives:

(36) $${\dot{p}}_{R}=2n\lambda^{+}\left[\begin{array}{l} f_{5}-f_{6}+f_{11}-f_{12}\\ f_{1}-f_{2}+f_{7}-f_{8}\\ -f_{3}+f_{4}-f_{9}+f_{10}\\ -\frac{3}{16mn^{3}}{\left\|p_{R}\right\|}_{2}^{2} \end{array}\right]$$

Equations (28) and (36) are ordinary differential equations about generalized coordinates $s_{R}$ and generalized momentum $p_{R}$, as well as the VDEs of the system. Equations (1), (2), (28), and (36) together constitute the IDEs of the sensor.

#### 3.2.2. Equation Solving

The motion of the measured object generally starts from rest. Therefore, based on Equations (3), (21) and (28), the initial value conditions of the IDEs can be obtained:

(37) $$\{\begin{array}{c} s_{R}^{\left(0\right)}={\left[\begin{array}{cccc} 0 & 0 & 0 & 1 \end{array}\right]}^{T}\\ p_{R}^{\left(0\right)}={\left[\begin{array}{cccc} 0 & 0 & 0 & 0 \end{array}\right]}^{T} \end{array}$$

where superscript (*N*) indicates the calculation time.

Replace the derivative of the current time with the average value of the derivative at two times before and after the current time in the classic trapezoidal formula, which can list the analytical formulas of the generalized coordinates $s_{R}$ and generalized momentum $p_{R}$ at each calculation time. The recurrence formula is shown as:

(38) $$Y^{\left(N+2\right)}=Y^{\left(N+1\right)}+\frac{h}{2}\left(3{\dot{Y}}^{\left(N+1\right)}-{\dot{Y}}^{\left(N\right)}\right)\left(N\geq 1\right)$$

where $ϒ={\left[\begin{array}{cc} {\left(s_{R}\right)}^{T} & {\left(p_{R}\right)}^{T} \end{array}\right]}^{T}$, *h* is the number of sampling nodes.

**Figure 5:**
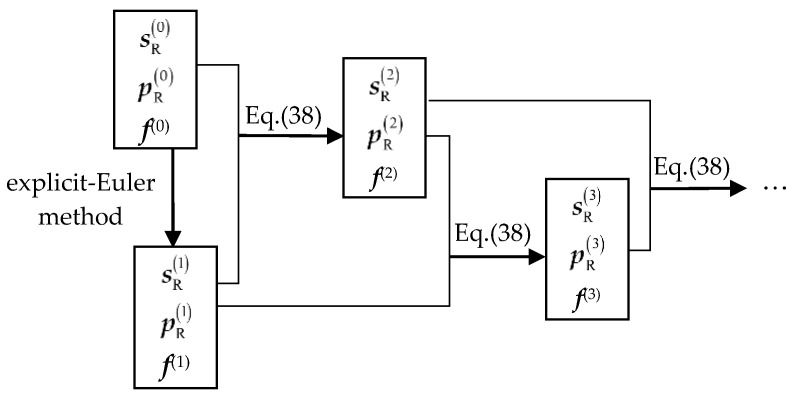
The basic structure of recursive expressions about generalized coordinates $s_{R}$ and generalized momentum $p_{R}$ display.

It is worth mentioning that Equation (38) needs to be started by the explicit Newton–Euler method. The recursive process of solving inverse dynamics is shown in Figure 5.

According to the calculation structure in Figure 5, the value of the generalized coordinate $s_{R}$ at each calculation time can be calculated. Then combine Equations (1), (2), and (3) to complete the calculation of the IDEs, that is, the decoupling of the acceleration.

## 4. Numerical Simulation

Up to now, there is no clear definition about the measurement error (solution error of the IDEs) of the six-axis accelerometer. This article defines its measurement error as linear acceleration measurement error and angular acceleration measurement error to improve the applicability of the sensor on different occasions.

(39) $$\{\begin{array}{c} \delta_{a}=\frac{1}{3}\left(\frac{\max({\bar{a}}_{x}-a_{x})}{\max(a_{x})-\min(a_{x})}+\frac{\max({\bar{a}}_{y}-a_{y})}{\max(a_{y})-\min(a_{y})}+\frac{\max({\bar{a}}_{z}-a_{z})}{\max(a_{z})-\min(a_{z})}\right)\times 100\%\\ \delta_{e}=\frac{1}{3}\left(\frac{\max({\bar{e}}_{x}-e_{x})}{\max(e_{x})-\min(e_{x})}+\frac{\max({\bar{e}}_{y}-e_{y})}{\max(e_{y})-\min(e_{y})}+\frac{\max({\bar{e}}_{z}-e_{z})}{\max(e_{z})-\min(e_{z})}\right)\times 100\% \end{array}$$

where ***a****_x_*, ***a****_y_*, ***a***_*z*_ are the vectors composed of the three components of ***a*** at all measurement times, and ***e****_x_*, ***e****_y_*, ***e***_*z*_ are the vectors composed of the three components of ***e*** at all measurement times, respectively. $\left(\bar{\cdot }\right)$, (·) are the measured value and theoretical value of acceleration respectively. It is worth mentioning that if there is a situation where the theoretical value is a constant, that is, the denominator in Equation (39) is zero, the denominator is taken as 1 to describe the drift error.

**Figure 6:**
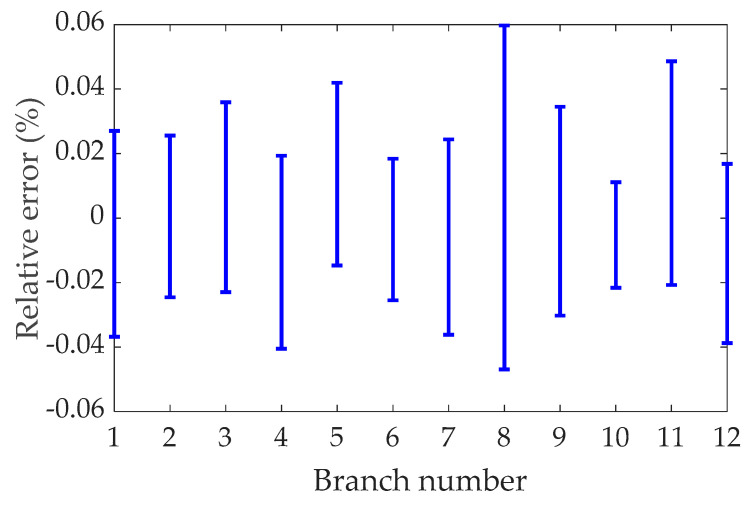
The relative error between the calculation result of the forward dynamic equations (FDEs) and the simulation result of ADAMS.

As a numerical example, the mass and side length of the inertial mass are set to 0.5 kg and 42 mm, respectively, and the initial length and stiffness of the branch chain are set to 24 mm and 2.07 × 10^5^ N/mm respectively. A set of accelerations of any given base, in which the linear acceleration and angular acceleration have a frequency of 5 Hz, and the amplitudes are 1.97 × 10^4^ mm/s^2^ and 5.17 × 10^2^ rad/s^2^, respectively. The difference between the calculated results of FDEs and ADAMS software simulation results was compared within 3 s. The range of relative errors of the axial forces of the 12 branches is shown in Figure 6, respectively, which verifies that the FDEs presented in this article is correct. Figure 6 shows that the relative error is less than 0.06%, indicating that the calculated values of FDEs are completely consistent with the theoretical values. It is worth noting that, in order to obtain the above-mentioned axial force of the branch, the time used for the calculation process of the FDEs and ADAMS simulation are 0.09 s and 24.17 s, respectively. Among them, the calculation time of the FDEs is obtained according to the ‘tic-toc’ command in the MATLAB software, and the simulation time of ADAMS is determined according to the time consumed by the ADAMS software simulation process recorded by the computer. For the above cases study, we use an Intel CORE I7-8750H @ 2.20 GHz CPU and a 8 GB RAM and MATLAB R2018a.

**Figure 7:**
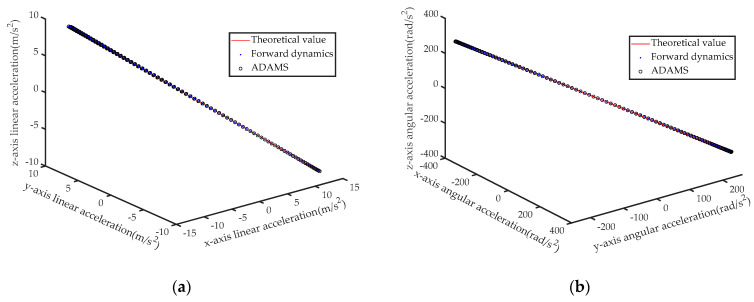
(**a**) Calculated and theoretical values of linear acceleration; (**b**) Calculated and theoretical values of angular acceleration.

Further, the IDEs of the six-axis accelerometer are solved based on the axial force obtained by the FDEs and the axial force obtained by the ADAMS simulation respectively, and the results are shown in Figure 7 respectively.

**Figure 8:**
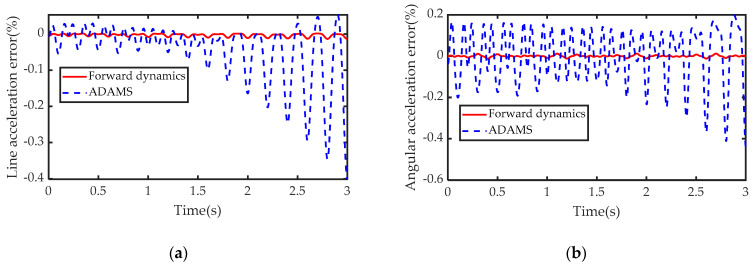
Numerical simulation error: (**a**) linear acceleration measurement error; (**b**) angular acceleration measurement error.

Then, combined with Equation (39) and Figure 7, the solution error and time of the IDEs of the sensor are shown in Figure 8 and Table 1. From the calculation results of the sensor’s IDEs, the FDEs is more accurate than ADAMS. This is because ADAMS solves differential equations through numerical recursive methods, while FDEs are algebraic equations without truncation errors. In addition, according to Table 1, the calculation time of the IDEs is far less than the measurement time, which indicates that this algorithm meets the real-time requirements.

## 5. Actual Experiment

An experimental prototype of the six-axis accelerometer is constructed to verify the validity of the dynamics mathematical model of the sensor. Figure 1b shows the physical prototype of the sensor used in this experiment, the main structural parameters of the physical prototype are given in Table 2.

**Figure 9:**
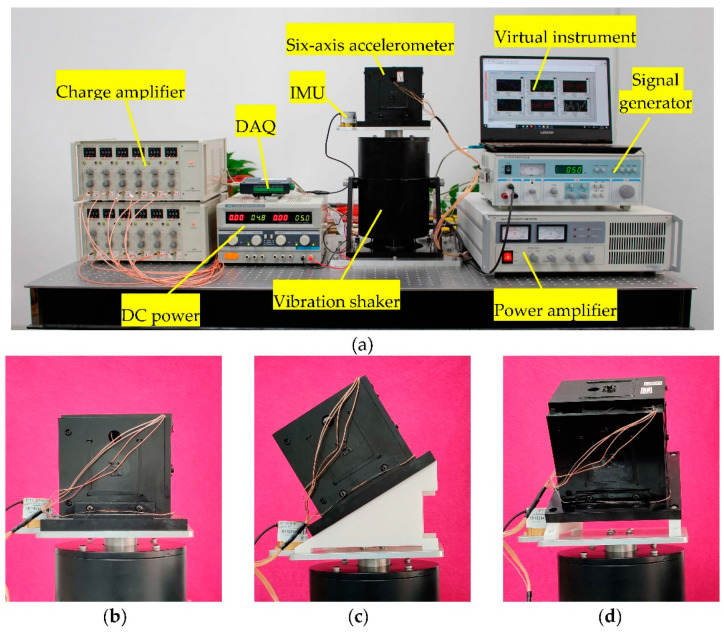
Experimental platform of six-axis accelerometers. (**a**) Arrangement of test instruments; (**b**) Measurement scheme 1; (**c**) Measurement scheme 2; (**d**) Measurement scheme 3.

The experimental platform is shown in Figure 9. The experimental platform and test scheme mainly include the following three modules.

***A***. Vibration shaker module. The experimental instruments in this module are composed of a signal generator, a power amplifier, and a vibration shaker. The signal and energy of the vibration shaker are provided by a signal generator and a power amplifier, respectively.

***B***. Sensor module. This part of the test instrument consists of a six-axis accelerometer, an IMU, and a DC power. The DC power is used to provide power to the IMU. IMU performance indicators are shown in Table 3. Both the IMU and the six-axis accelerometer are used to measure the vibration of the vibration shaker, and the IMU measurement results are regarded as standard values. The accuracy of the measurement results of the sensor in this article is verified by comparing the measurement results of the six-axis accelerometer and IMU.

***C***. Data acquisition and processing module. This module is composed of a charge amplifier, a data acquisition card (DAQ, the allowable maximum sampling rate is 200 KHz), and a computer (used to display virtual instruments based on the software LabVIEW). On the one hand, the data in the DAQ is displayed and saved by the virtual instrument in the computer. On the other hand, the data measured by the IMU is also saved by the computer.

### 5.1. Actual Experiment 1

According to the measurement scheme in Figure 9b–d, we set up the following test plan to verify the accuracy of this theoretical model.

(1). Set the excitation frequency (*f_m_*) and amplitude (*A_m_*) of the vibration shaker of the calibration platform shown in Figure 9 to 5 Hz and 5 mm, and the sampling time in the virtual instrument to 60 s. Six sets of measurement data are obtained by setting the sampling frequency in the virtual instrument to 500 Hz, 600 Hz, 700 Hz, 800 Hz, 900 Hz and 1000 Hz, respectively. Without the loss of generality, the maximum relative error *δ*_f1max_ and the minimum relative error *δ*_f1min_ (compare with the calculated results of FDEs) of the measurement signal of the first branch chain are shown in Table 4. Furthermore, substituting the data into the IDEs of this article to decouple the acceleration, according to Equation (39), the calculation error of acceleration is shown in Table 4.

According to Table 4, the calculation accuracy of the FDEs constructed in this article is higher than that in Reference [33], and the calculation accuracy and efficiency of the IDEs are also better than the modeling method in Reference [31].

(2). On the basis of (1), we set the test sampling frequency to 1000 Hz, and changed the frequency and amplitude of the vibration shaker respectively. The test results are shown in Table 5.

**Figure 10:**
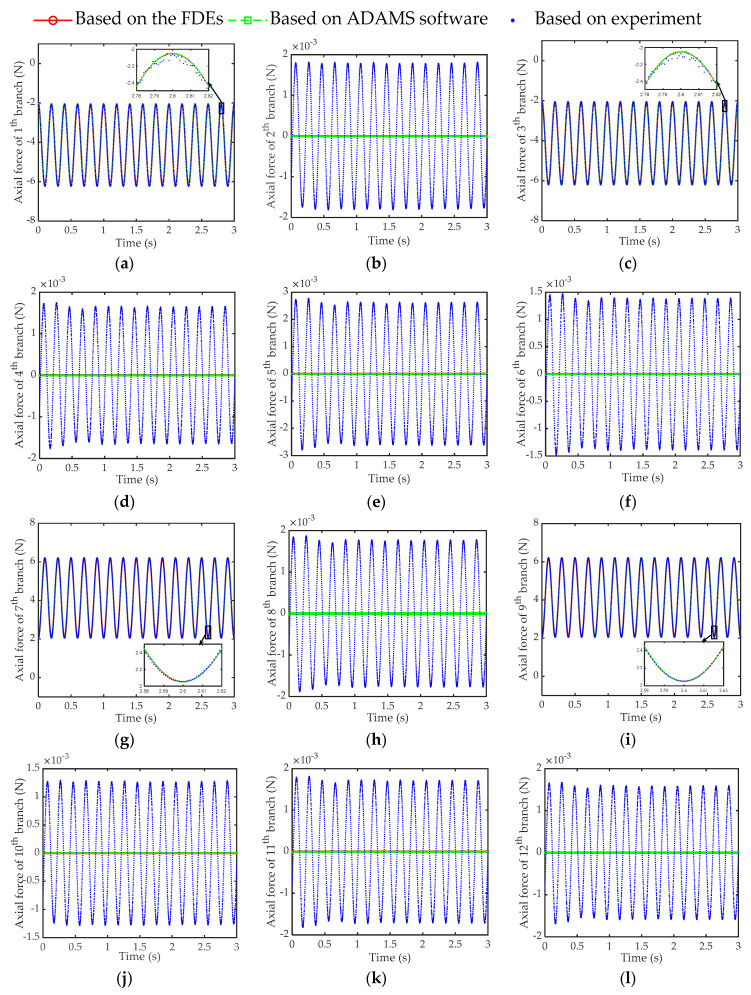
Comparison diagram of axial force calculated by three methods. (**a**) 1st branch; (**b**) 2nd branch; (**c**) 3rd branch; (**d**) 4th branch; (**e**) 5th branch; (**f**) 6th branch; (**g**) 7th branch; (**h**) 8th branch; (**i**) 9th branch; (**j**) 10th branch; (**k**) 11th branch; (**l**) 12th branch.

Furthermore, according to the parameter settings in Table 2 and the above-mentioned test conditions, the parameters are substituted into the FDEs of this article, and a virtual prototype with the same parameters is established in the ADAMS software. Taking the sampling frequency of 1000 Hz in Table 4 as an example, the results of the axial force of the 12 branch chains obtained based on three methods (FDEs of the sensor, simulation of ADAMS software, and the experimental measurement) are shown in Figure 10. Among them, the curve of the calculation result of the FDEs almost coincides with the curve of the simulation result of the ADAMS software.

**Figure 11:**
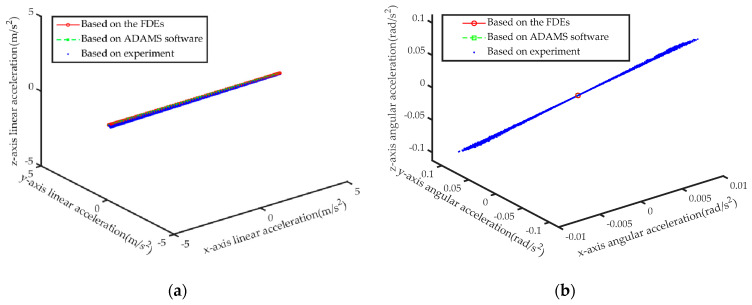
Comparison of the calculation results of the IDEs based on the results of the three methods. (**a**) Linear acceleration; (**b**) Angular acceleration.

**Figure 12:**
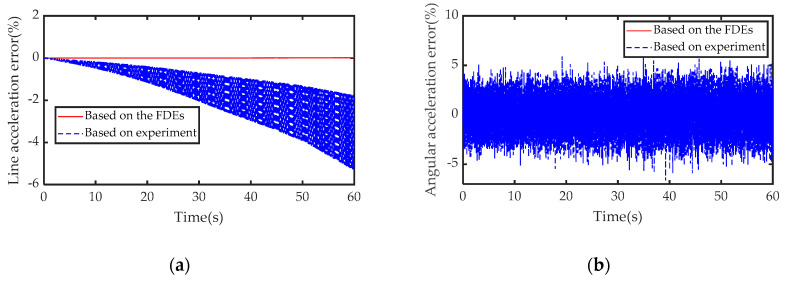
Test error: (**a**) linear acceleration measurement error; (**b**) angular acceleration measurement error.

Substituting the axial forces obtained by the three methods in Figure 10 into the IDEs of this article, the calculation results of acceleration are shown in Figure 11, respectively. Then, based on Figure 11, combining Equation (39) with the measurement result of IMU, the solution error of the IDEs of the sensor is shown in Figure 12.

Based on Table 4 and Table 5 and Figure 10 and Figure 12, we can verify the correctness of the mathematical model of FDEs and IDEs constructed in this article.

### 5.2. Actual Experiment 2

**Figure 13:**
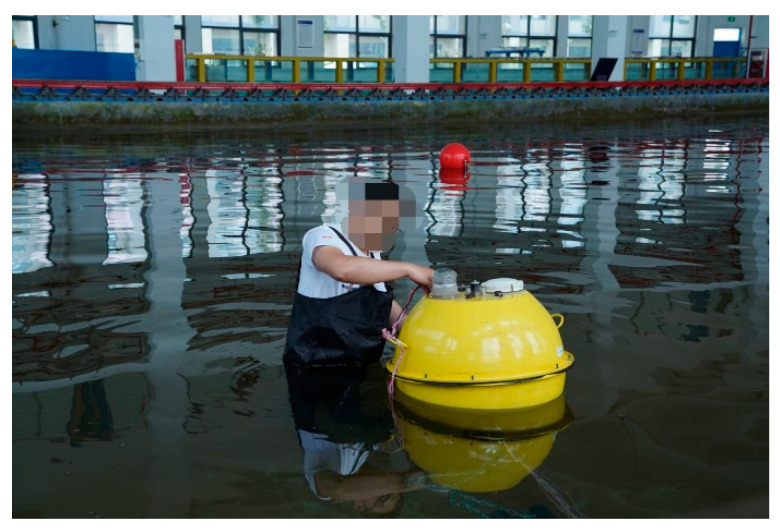
Wave pool experiment.

In order to further verify the applicability of the model constructed in this article, we apply the six-axis accelerometer to ocean wave buoy to measure the height of waves [43], as shown in Figure 13.

The acceleration of the buoy can be calculated according to the test data and the IDEs of this article, and then the height of the wave can be calculated by referring the acceleration-displacement integral algorithm of Reference [43]. The results of the test are shown in Table 6. Comparing the test data with the measurement results of TRIAXYS wave buoy, the relative errors of the measurement schemes in this article are shown in Table 6.

The results in Table 6 show that the dynamic equations studied in this article has a certain general applicability.

## 6. Conclusions and Discussion

In this work, a mathematical model of the dynamics of the sensor is established. The results of the virtual and actual experiments demonstrate the accuracy of the dynamic model and the practicability of the six-axis accelerometer proposed in this article. The main conclusions are as follows:

(1) The analytical expression of the equivalent length of the branch chain with respect to the relative pose parameters between {O_2_} and to {O_1_} is obtained by analyzing the kinematic equation of the branch chain and combining with the Taylor formula. On the premise of avoiding the forward kinematics of the sensor elastic body, the coordination equation between the branch chain lengths is obtained based on the expression of the branch chain length. Based on this, combined with the Newton–Euler equation of the system, the analytical expression of the axial force of the branch chain with respect to the measured acceleration is obtained, which comprise the FDEs of the sensor. The results of FDEs are compared with those of the simulation and experimental, and their relative errors are less than 0.06% and 2.21% respectively. This demonstrates that the modeling scheme and experimental scheme in this article are correct. This lays a theoretical foundation for the calibration, fault diagnosis and structural optimization of multi-dimensional sensors.
(2) The Routh equation can be used to establish the differential equations of motion when the system has dependent coordinates. The Hamiltonian equations of the system are related to generalized momentum and generalized velocity. The undetermined multiplier in the equation is related to the mass of the inertial mass, the side length of the inertial mass, the axial force of the branch chain and the generalized momentum. The Legendre transformation and the analytical solution of the undetermined multiplier can be used to derive the VDEs of the system. The IDEs of the system include VDEs and Newton–Euler equations. Based on the orthogonal relationship between generalized coordinates and generalized momentum, the explicit recursive algorithm of the unknown quantity in the IDEs can be given. The actual prototype experiment shows that the relative errors of linear acceleration and angular acceleration are 6.53% and 7.65%, respectively. Also, the decoupling algorithm meets the real-time requirements. The test accuracy and efficiency are better than the performance test of the physical prototype of the same type of six-axis accelerometer [26,41]. The relative error of the ocean wave buoy test based on the IDEs of the sensor does not exceed 8.20%, which demonstrates the universal applicability of the scheme proposed in this article.

The process and analysis of dynamic equations could possibly provide readers a reference for the practical application of six-axis accelerometer based on the parallel mechanism. The research also provides inspiration for our future work. Although the feasibility of the measurement scheme measured of the sensor has been verified by the vibration shaker, a more advanced calibration platform is needed for calibration research. Therefore, we will design a calibration platform for a six-axis accelerometer that can achieve six-degree-of-freedom motion to further verify the theoretical model of the sensor.

## Figures and Tables

**Table 1:** The solution result of the IDEs of the sensor.

Types of Axial Force	Linear Acceleration Error *δa*/%	Angular Acceleration Error *δ_e_*/%	Calculating Time *t*/s
Calculation result of the FDEs	0.014	0.013	0.037
ADAMS simulation	0.401	0.442	0.037

**Table 2:** Main physical parameters of the physical prototyping.

Component	Material	Mass/g	Dimensions/mm
Inertial Mass	45# Steel	1450.00	60 × 60 × 60
Flexible Spherical Joint	65 Mn	3.18	
Locking Plate	6063 Duralumin	0.23	
Pretension Rod	45# Steel	10.60	
Sub-pedestal	6063 Duralumin	113.42	
Pedestal	6063 Duralumin	846.20	
Piezoelectric Ceramic	Type: YT-5L	1.12	*Φ*8 × 3

**Table 3:** Inertial measurement unit (IMU) performance indicators.

Performance	Value	Performance	Value
Range: roll, pitch, yaw (°/s)	±300	Range: X, Y, Z (g)	±6
Zero error (°/s)	<0.5	Linear acceleration resolution (g)	<0.001
Zero instability (°/s)	6	Bias stability (g)	<0.007
Angular velocity resolution (°/s)	0.01	Measurement bandwidth (Hz)	20

**Table 4:** Test results of the physical prototype of the sensor.

Sampling Frequency/Hz	*δ*_f1max_/%		*δ*_f1min_/%		*δa*/%		*δ_e_*/%		*t*/s	
	This Work	Ref. [33]	This Work	Ref. [33]	This Work	Ref. [31]	This Work	Ref. [31]	This Work	Ref. [31]
500	2.21	2.53	−2.15	−2.79	6.53	9.35	7.65	9.65	1.03	3.33
600	1.97	2.21	−1.81	−2.52	6.07	9.12	7.18	9.31	1.24	3.91
700	1.73	1.93	−1.76	−2.34	5.77	8.83	6.97	8.92	1.65	4.67
800	1.55	1.57	−1.99	−2.17	5.45	8.74	6.64	8.79	2.04	5.27
900	1.24	1.45	−1.81	−1.87	5.45	8.79	6.68	8.14	2.63	5.82
1000	1.08	1.37	−1.57	−1.65	5.30	8.43	6.64	8.62	3.15	6.48

**Table 5:** Test results of the physical prototype of the sensor.

Setting of Test Parameters		*δ*_f1max_/%	*δ*_f1min_/%	*δa*/%	*δ_e_*/%	*t*/s
Vibration frequency of the vibration shaker (*A_m_* = 5 mm)	6	1.23	−1.42	6.25	7.37	3.12
	7	1.11	−1.61	6.93	6.84	3.19
	8	1.09	−1.59	6.36	7.51	3.21
	9	1.23	−1.63	7.24	7.79	3.15
	10	1.19	−1.71	6.68	7.63	3.17
Vibration amplitude of the vibration shaker (*f_m_* = 5 Hz)	6	1.02	−1.47	6.75	6.72	3.14
	7	1.13	−1.56	6.23	7.18	3.19
	8	1.21	−1.49	7.11	7.39	3.18
	9	0.97	−1.38	6.21	7.16	3.13
	10	1.06	−1.62	6.47	6.79	3.18

**Table 6:** Experimental results of wave height.

Measuring Moment (h)	0:00	2:00	4:00	6:00	8:00	10:00	12:00	14:00	16:00	18:00	20:00	22:00
Based on this work (m)	0.53	0.36	0.34	0.38	0.41	0.56	0.59	0.42	0.36	0.49	0.38	0.40
Based on TRIAXYS wave buoy (m)	0.49	0.35	0.34	0.36	0.42	0.61	0.56	0.40	0.39	0.47	0.41	0.37
Relative error (%)	8.16	2.86	0	5.56	2.38	8.20	5.36	5.00	7.69	4.26	7.32	8.11

## Data Availability

No new data were created or analyzed in this study. Data sharing is not applicable to this article.
